# Montelukast Drug May Improve COVID-19 Prognosis: A Review of Evidence

**DOI:** 10.3389/fphar.2020.01344

**Published:** 2020-09-04

**Authors:** Jean Barré, Jean-Marc Sabatier, Cédric Annweiler

**Affiliations:** ^1^ Department of Geriatric Medicine and Memory Clinic, Research Center on Autonomy and Longevity, University Hospital, Angers, France; ^2^ Aix-Marseille University, Institute of NeuroPhysiopathology, UMR 7051, Marseille, France; ^3^ UPRES EA 4638, Université d’Angers, Angers, France; ^4^ Department of Medical Biophysics, Schulich School of Medicine and Dentistry, Robarts Research Institute, the University of Western Ontario, London, ON, Canada

**Keywords:** coronavirus disease 2019, severe acute respiratory syndrome coronavirus 2, montelukast, lukasts, treatment, research

## Abstract

With the lack of effective therapy, chemoprevention and vaccination, focusing on the immediate repurposing of existing drugs gives hope of curbing the pandemic. Interestingly, montelukast, a drug usually used in asthma, may be proposed as a potential adjuvant therapy in COVID-19. The aim of the present article was to review the properties of montelukast that could be beneficial in COVID-19. Ten experimentally supported properties were retrieved, either related to SARS-CoV-2 (antiviral properties, prevention of endotheliitis and of neurological disorders linked to SARS-CoV-2), and/or related to the host (improvement of atherogenic vascular inflammation, limitation of the ischemia/reperfusion phenomenon, improvement of respiratory symptoms), and/or related to serious COVID-19 outcomes (limitation of the cytokine storm, mitigation of acute respiratory distress syndrome), and/or related to tissue sequelae (antioxidant properties, anti-fibrosis effects). Based on gathered theoretical evidence, we argue that montelukast should be further tested to prevent and treat COVID-19 outcomes.

## Introduction

Coronaviruses are a large family of single-stranded RNA viruses, which infect animals and humans. Since December 2019, the coronavirus disease 2019 (COVID-19) caused by the severe acute respiratory syndrome coronavirus 2 (SARS-CoV-2; previously 2019-nCoV) is spreading worldwide. The virus is primarily spread between people during close contact, most often *via* small droplets produced by coughing, sneezing, and talking. COVID-19 is characterized by fever, cough, severe pneumonia, RNAaemia, combined with the incidence of ground-glass opacities, clot formation and endotheliitis, and a variety of clinical signs including fatigue, cardiac and neurological outcomes (Ahn et al., 2020). Of note, while the majority of cases result in only mild symptoms, some progress to acute respiratory distress syndrome (ARDS) possibly precipitated by significant increase in blood levels of cytokines and chemokines. This “cytokine storm”, reportedly due to angiotensin-converting enzyme-2 (ACE2) downregulation by SARS-CoV-2 (Bourgonje et al., 2020), triggers a proinflammatory environment which is strongly associated with severe tissue damages, contributing to ARDS and fatal outcomes of COVID-19 patients (Kimura et al., 2013).

As of June 2020, the COVID-19 pandemic has affected millions of people in 196 countries and left hundreds of thousands dead. With the lack of effective therapy, chemoprevention and vaccination, focusing on the immediate repurposing of existing drugs gives hope of curbing the pandemic. Interestingly, a recent *in silico* exploration identified montelukast (MK), from the Leukasts family (LKs; i.e. cysteinyl leukotriene receptors antagonists), among the top-scoring clinically-oriented drugs likely to inhibit SARS-CoV-2 main protease (Huynh et al., 2020). One retrospective study consistently found that older asthmatic outpatients receiving MK had fewer episodes of confirmed COVID-19 than those not using MK (Bozek and Winterstein, 2020). The aim of this article was to review the properties of LKs, especially of MK, that could be beneficial in COVID-19 and would deserve further dedicated studies.

## Montelukast

MK works as a cysteinyl leukotriene (cysLT) receptor antagonist. Leukotrienes are inflammatory mediators produced by the immune system. They promote bronchoconstriction, inflammation, microvascular permeability, and mucus secretion in asthma and chronic obstructive pulmonary disease. Consequently, use of high-dose MK as an anti-inflammatory agent is effective in acute asthma (Wu et al., 2003). MK is mainly used as a complementary therapy in adults in addition to inhaled corticosteroids. The use of MK is also known to decrease the frequency and severity of wheezing after an upper respiratory tract infection caused by adenovirus, influenza, metapneumovirus or coronavirus (Brodlie et al., 2015). Common side effects include diarrhea, nausea, vomiting, mild rashes, asymptomatic elevations in liver enzymes and fever. In 2019 and 2020, concerns for neuropsychiatric reactions were added to the label in the UK and US where the most frequently suspected were nightmares, depression, insomnia, aggression, anxiety and abnormal behavior (Glockler-Lauf et al., 2019).

**Figure 1:**
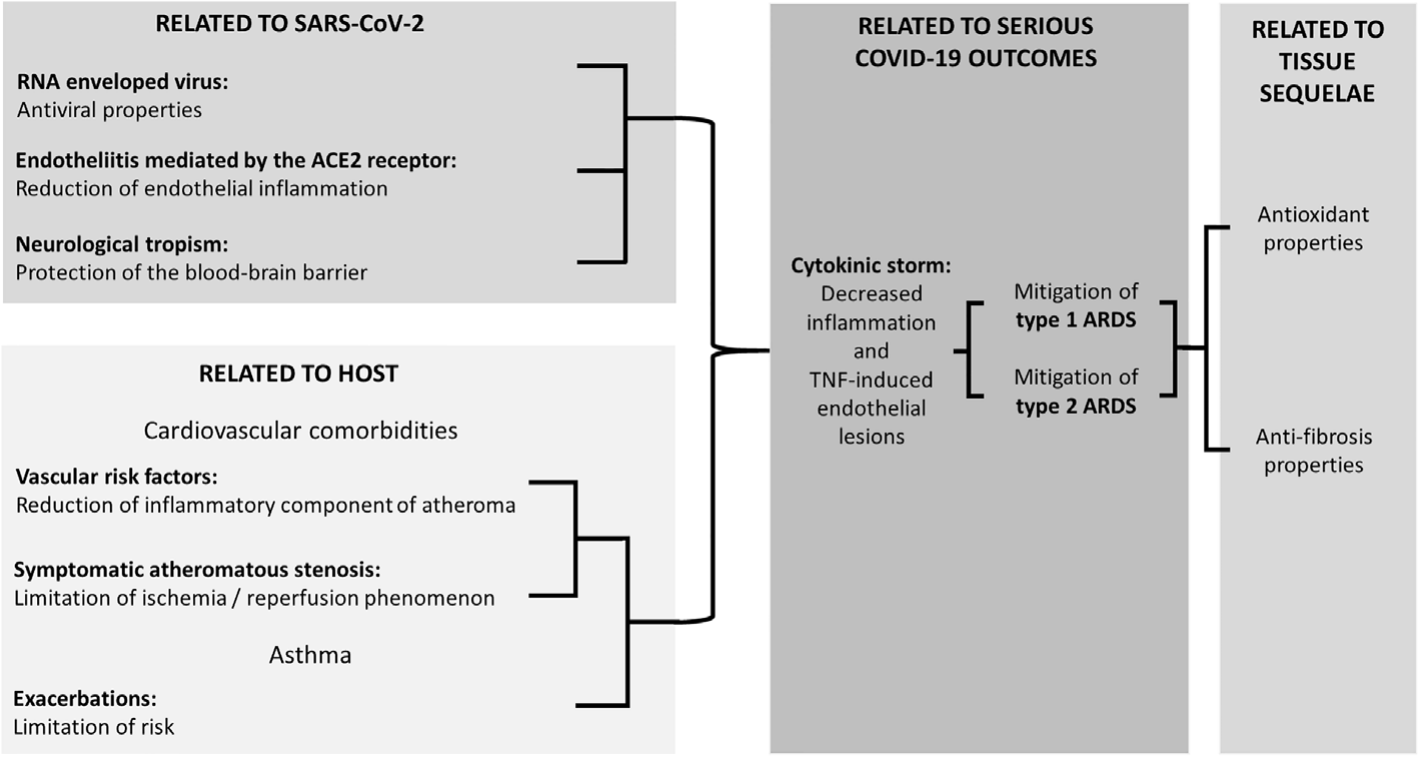
Experimentally supported properties of Cyst LT1 receptor antagonists potentially beneficial in COVID-19.

Apart from MK, LKs also include Zafirlukast (ZK) and pranlukast (PK). These three compounds may have properties of potential interest to treat COVID-19, the main ones of which are illustrated in **Figure 1** and described hereafter.

## Properties of MK Related to SARS-CoV-2

### Antiviral Properties

Several antiviral properties of MK, potentially useful in COVID-19, have been described *in vitro* and *in vivo*, based on distinct mechanisms depending on the virus under investigation. For *Influenzae A* virus, an inhibition of the expression of the viral genome was observed with MK (Landeras-Bueno et al., 2016). For *flaviviridae*, in particular Zika virus, an irreversible and early inactivation of the virus was reported, probably due to some damage to the lipid membrane (Chen et al., 2020). Three distinct mechanisms were proposed to support the beneficial impact of MK on Zika virus: i) a direct antiviral action, ii) an antagonization of the cytokine storm, and iii) an inhibition of the vertical transmission by a MK-related neuroprotective effect on the brain of fetus. For the hepatitis C virus, MK induced a dose-dependent decrease in the levels of RNAs expressed, indicating an inhibition of viral replication (Ruiz et al., 2020). MK also attenuated the initial responses to respiratory syncytial virus (RSV) infection in neonate and adult mice, and reduced the consequences of RSV reinfection in mice initially infected as neonates (Han et al., 2010; Kloepfer et al., 2011). Finally, in humans, Morita et al. (2017) have reported a decrease of almost 50% in the number of colds in younger boys aged 1 to 5.

### Endotheliitis Induced by SARS-CoV-2 Infection

SARS-CoV-2 interacts with the ACE2 receptors to infect the host (Bourgonje et al., 2020). This process is thought to promote the development of endotheliitis (Varga et al., 2020), a condition that may be responsible for the multiplicity of clinical signs observed in COVID-19. MK antagonizes the inflammatory cascade induced by angiotensin II in vascular smooth muscle cells (Mueller et al., 2010) and could therefore constitute a specific treatment for the inflammation induced by this condition (Fidan and Aydoğdu, 2020).

### Neurological Disorders Induced by SARS-CoV-2 Infection

Central nervous system disorders affect ca. 36.4% of patients with COVID-19 (Mao et al., 2020), mainly involving anosmia, dysgeusia, and headache. More serious manifestations such as seizures, delirium, encephalitis, and stroke have also been reported (Mao et al., 2020). LK limits the damage induced on the blood-brain barrier and has shown anti-convulsant properties in an experimental animal model of epilepsy (Lenz et al., 2014). Such protection of the blood-brain barrier could limit the occurrence and severity of brain damage (Zhou L. et al., 2019). It was also reported that MK improves fiber re-organization and long-term functional recovery after brain ischemia, enhancing recruitment and maturation of oligodendrocyte precursor cells (Gelosa et al., 2019). Additionally, a 6-week treatment with MK reduced neuroinflammation and elevated hippocampal neurogenesis through inhibition of the GPR17 receptor in younger and older rats (Marschallinger et al., 2015), with potential benefits for the prevention of manifestations such as delirium.

## Properties of MK Related the Host

### Atherogenic Vascular Inflammation

It has been proposed that some severe complications of COVID-19 are mainly related to the host (Zhang et al., 2020). They are influenced by the age, gender and comorbidities, notably linked to preexisting inflammatory vascular and respiratory conditions. The cysLT are precisely strongly involved in the inflammatory phase of the atheromatous process although they are not used in this indication thus far. Antagonization of cysLT receptors greatly attenuates arterial spasm on human coronary arteries with atherosclerotic lesions, but it has no effect on healthy coronary arteries (Allen et al., 1993). A systematic review of the anti-atheromatous properties of MK in twenty-six animal and two human studies concluded that all studies supported the efficacy of LKs and MK on the atheromatous process (Hoxha et al., 2018). LKs could therefore reduce COVID-19 mortality in atheromatous patients, conferring a protection that would be (theoretically) proportional to the extent and severity of the atheromatous lesions (Almerie and Kerrigan, 2020).

### Ischemia/Reperfusion

The ischemia/reperfusion phenomenon results in downstream vascular lesions following reperfusion. In patients with severe atheromatous disease, tissue hypoxia and hypoperfusion increase the risk of developing new endothelial lesions and ruptured atheroma plaque, inducing thrombosis and emboli. This may explain in part why COVID-19 is associated with an increased risk of arterial and venous thromboembolism, which affects approximately 30% of SARS-CoV-2-infected patients hospitalized in intensive care units (Klok et al., 2020). MK alleviates the ischemia/reperfusion phenomenon in animal models of intestinal anastomosis (Sayin et al., 2020), in skeletal muscles (Bilgiç et al., 2018), in the spinal cord (Korkmaz et al., 2015), and even following ovarian (Oral et al., 2011) or testicular torsion/distortion (Sılay et al., 2014). A coronary stent coated with MK particles is being developed (Zamani et al., 2016).

### Asthma, Hyper-Reactivity Bronchitis, and Post-Infectious Cough

Asthma, for which LKs are usually prescribed, is a frequent and serious condition affecting 7%–8% of the population, though it is still under-diagnosed and under-treated (Deschildre, 2014). MK is effective against cough when it is an asthmatic equivalent, regardless of the functional respiratory parameters (Miwa et al., 2018). In contrast, MK has not shown any efficacy in chronic post-infectious cough (Wang et al., 2014), even though there was a high level of subjective improvement in the placebo group in this study (Wang et al., 2014). It would be of interest to examine MK on the mild symptomatic forms of COVID-19 respiratory damage (bronchospasms, cough, and chest pain).

## Properties of MK Related to COVID-19 Serious Outcomes

### Cytokine Storm

The cytokine storm, corresponding to an unopposed generation of both pro-inflammatory and anti-inflammatory cytokines by the innate immune system, is responsible for most of the serious pulmonary complications of COVID-19 (Russell et al., 2020). The antagonist action of ZK on CystLT1 receptor protects the endothelium from inflammatory lesions induced by TNF-α (Zhou X. et al., 2019). By increasing IFN-γ production and inhibiting the expression of cytokines such as IL-1β, IL-6, and IL-8, the inflammatory chain-reaction could be better controlled (Han et al., 2010). Clinically, MK is used to reduce drug-related cytokine reactions induced by daratumumab (Chari et al., 2018) and rituximab (Kotchetkov et al., 2020). In this indication, MK is associated with a marked decrease in frequency and intensity of cytokine reactions and this action seems to be strengthened by the addition of an anti-H1, namely rupatadine (Kotchetkov et al., 2020).

### Acute Respiratory Distress Syndrome

SARS-CoV-2-infected patients classically show mild symptoms that may gradually progress to more severe manifestations such as lethal ARDS. The type 1 ARDS is secondary to a direct alveolar inflammatory reaction, whereas the type 2 ARDS is secondary to systemic damage and occurs in the context of multi-visceral failure. To date, there is no effective chemotherapeutic treatment for ARDS. The cornerstone of this condition remains the mechanical ventilation (Fan et al., 2018).

Regarding the type 1 ARDS, LK showed significant benefit on models induced by inhalation of irritant product like chlorine (Hamamoto et al., 2017) or pro-inflammatory lipids (Aquino-Junior et al., 2019), with a decrease in the intensity of the induced cytokine cascade and a lesser activation of neutrophils in the bronchoalveolar fluid. A similar effect was also reported in an animal model of malignant flu (Cardani et al., 2017).

Regarding the type 2 ARDS in an animal model of lung lesions induced by hepatic ischemia (Yeh et al., 2015) or hemorrhagic shock (Al-Amran et al., 2013), administration of LK resulted in a pulmonary reduction of neutrophil infiltration, lung inflammation, oxidative stress, and extent of lesions, along with a significant decrease in TNF-α and IL-6 cytokines in the pulmonary parenchyma and bronchoalveolar lavage.

## Properties of MK Related to Tissue Sequelae

### Antioxidant Properties

An overproduction of reactive oxygen species (ROS) is crucial for viral replication and the subsequent virus-associated disease (Khomich et al., 2018). Experimental animal models of ARDS have shown enhanced ROS levels and disturbance of antioxidant defense during SARS-CoV infection (van den Brand et al., 2014). In cells infected with SARS-CoV, there was a greater amount of activated (phosphorylated) forms of all mitogen-activated protein kinase (MAPK) members (Khomich et al., 2018); i.e. a family of serine/threonine that are activated in response to environmental stresses including oxidative stress, DNA damage, carcinogenic stimuli and viral infections. Clinically, Shao et al. (2006) observed an upregulation of mitochondrial genes and genes responding to oxidative stress in peripheral blood mononuclear cells of convalescent SARS-CoV patients. Some of these genes, including PRDX1, FTH1 and FOS, are sensitive to oxidative stress and showed a remarkable elevation. These results support a role for oxidative stress during COVID-19. Importantly, protective effects of MK are not limited to inflammatory and microbial infectious attacks, but also include protection against chemotoxicity (bleomycin, cysplatin, doxorubicin, statin, paracetamol) (Hareedy et al., 2019) and radiotoxicity (Hormati et al., 2020) in animal experiments, which demonstrates some antioxidant properties resulting in increased mitochondrial mass and functionality, together with increased intracellular cyclic adenosine 3, 5′-monophosphate (cAMP) level and activation of the Krebs cycle (Wang et al., 2019).

### Anti-Fibrosis Properties

Using MK may limit the residual extent of COVID-19 sequelae of pulmonary fibrosis, as for scar formation after lung surgery (Peng et al., 2017). MK regulates the extracellular remodeling matrix and inhibits the formation of fibrosis (Debelleix et al., 2018). This anti-fibrotic potential has been confirmed in an animal model of pulmonary fibrosis induced by bleomycin (Topaloğlu et al., 2018). Similarly, a recent meta-analysis confirmed in women that MK decreases the risk of retractile fibrosis after the placement of a silicone implant in breast reconstruction surgery (Wang et al., 2020).

## Conclusions

Although quantity is not quality, these 10 effects of MK may constitute as many synergistic and potentiating therapeutic possibilities in COVID-19. MK is a commonly used drug that does not require any prior cardiological or biological examination; it can be prescribed for pregnant women and frail older adults, and it shows a “comfortable” therapeutic range. Moreover, it could be all the more effective for patients with comorbidities such as diabetes, sleep apnea, smoking, obesity, or symptomatic atherosclerotic lesions. We support the conduct of clinical trials testing the effect of MK in COVID-19 patients from a variety of populations, while keeping in mind its adverse effects. Finally, it should also be emphasized that a potential massive use of MK in COVID-19 would risk depriving asthma patients of their treatment, which should also be anticipated.

## Author Contributions

CA has full access to all of the data in the study, takes responsibility for the data, the analyses and interpretation and has the right to publish any and all data, separate and apart from the attitudes of the sponsors. All authors contributed to the article and approved the submitted version. Study concept and design: JB, J-MS, and CA. Drafting of the manuscript: JB and CA. Critical revision of the manuscript for important intellectual content: J-MS. Study supervision: CA.

## Conflict of Interest

The authors declare that the research was conducted in the absence of any commercial or financial relationships that could be construed as a potential conflict of interest.

## References

[B1] Ahn D. G. Shin H. J. Kim M. H. Lee S. Kim H. S. Myoung J. (2020). Current Status of Epidemiology, Diagnosis, Therapeutics, and Vaccines for Novel Coronavirus Disease 2019 (COVID-19). J. Microbiol. Biotechnol. 30, 313–324. 10.4014/jmb.2003.03011 32238757 PMC9728410

[B2] Al-Amran F. G. Hadi N. R. Hashim A. M. (2013). Cysteinyl leukotriene receptor antagonist montelukast ameliorates acute lung injury following haemorrhagic shock in rats. Eur. J. Cardiothorac. Surg. 43, 421–427. 10.1093/ejcts/ezs312 22851661

[B3] Allen S. P. Dashwood M. R. Chester A. H. Tadjkarimi S. Collins M. Piper P. J. (1993). Influence of Atherosclerosis on the Vascular Reactivity of Isolated Human Epicardial Coronary Arteries to Leukotriene C4. Cardioscience 4, 47–54. 8471741

[B4] Almerie M. Q. Kerrigan D. D. (2020). The association between obesity and poor outcome after COVID-19 indicates a potential therapeutic role for montelukast. Med. Hypotheses 143, 109883. 10.1016/j.mehy.2020.109883 32492562 PMC7255216

[B5] Aquino-Junior J. C. J. Brito A. A. Rigonato-Oliveira N. C. Damaceno-Rodrigues N. R. Oliveira A. P. L. Silva A. P. (2019). Montelukast, Leukotriene Inhibitor, Reduces LPS-Induced Acute Lung Inflammation and Human Neutrophil Activation. Arch. Bronconeumol. 55, 573–580. 10.1016/j.arbres.2019.05.003 31257011

[B6] Bilgiç M.İ. Altun G. Çakıcı H. Gideroğlu K. Saka G. (2018). The protective effect of Montelukast against skeletal muscle ischemia reperfusion injury: An experimental rat model. Ulus. Travma. Acil. Cerrahi. Derg. 24, 185–190. 10.5505/tjtes.2017.22208 29786827

[B7] Bourgonje A. R. Abdulle A. E. Timens W. Hillebrands J. L. Navis G. J. Gordijn S. J. (2020). Angiotensin-converting enzyme-2 (ACE2), SARS-CoV-2 and Pathophysiology of Coronavirus Disease 2019 (COVID-19). J. Pathol. 251, 228–248. 10.1002/path.5471 32418199 PMC7276767

[B8] Bozek A. Winterstein J. (2020). Montelukast’s ability to fight COVID-19 infection. J. Asthma. 1–2. 10.1080/02770903.2020.1786112 32586154

[B9] Brodlie M. Gupta A. Rodriguez-Martinez C. E. Castro-Rodriguez J. A. Ducharme F. M. McKean M. C. (2015). Leukotriene receptor antagonists as maintenance and intermittent therapy for episodic viral wheeze in children. Cochr. Database. Syst. Rev. 2015, CD008202. 10.1002/14651858.CD008202.pub2 PMC6986470 26482324

[B10] Cardani A. Boulton A. Kim T. S. Braciale T. J. (2017). Alveolar Macrophages Prevent Lethal Influenza Pneumonia By Inhibiting Infection Of Type-1 Alveolar Epithelial Cells. PLoS Pathog. 13, e1006140. 10.1371/journal.ppat.1006140 28085958 PMC5268648

[B11] Chari A. Lonial S. Mark T. M. Krishnan A. Y. Stockerl-Goldstein K. E. Usmani S. Z. (2018). Results of an early access treatment protocol of daratumumab in United States patients with relapsed or refractory multiple myeloma. Cancer 124, 4342–4349. 10.1002/cncr.31706 30395359 PMC6587745

[B12] Chen Y. Li Y. Wang X. Zou P. (2020). Montelukast, an Anti-asthmatic Drug, Inhibits Zika Virus Infection by Disrupting Viral Integrity. Front. Microbiol. 10:3079. 10.3389/fmicb.2019.03079 32082265 PMC7002393

[B13] Debelleix S. Siao-Him Fa V. Begueret H. Berger P. Kamaev A. Ousova O. (2018). Montelukast Reverses Airway Remodeling in Actively Sensitized Young Mice. Pediatr. Pulmonol. 53, 701–709. 10.1002/ppul.23980 29493871

[B14] Deschildre A. (2014). Exacerbations of Asthma: A Demonstration That Guidelines Are Insufficiently Followed. Rev. Mal. Respir. 31, 1–3. 10.1016/j.rmr.2014.01.001 24461434

[B15] Fan E. Brodie D. Slutsky A. S. (2018). Acute Respiratory Distress Syndrome: Advances in Diagnosis and Treatment. JAMA 319, 698–710. 10.1001/jama.2017.21907 29466596

[B16] Fidan C. Aydoğdu A. (2020). As a Potential Treatment of COVID-19: Montelukast. Med. Hypotheses 142, 109828. 10.1016/j.mehy.2020.109828 32416408 PMC7211747

[B17] Gelosa P. Bonfanti E. Castiglioni L. Delgado-Garcia J. M. Gruart A. Fontana L. (2019). Improvement of fiber connectivity and functional recovery after stroke by montelukast, an available and safe anti-asthmatic drug. Pharmacol. Res. 142, 223–236. 10.1016/j.phrs.2019.02.025 30818044

[B18] Glockler-Lauf S. D. Finkelstein Y. Zhu J. Feldman L. Y. To T. J. (2019). Montelukast and Neuropsychiatric Events in Children with Asthma: A Nested Case-Control Study. J. Pediatr 209, 176–182.e4. 10.1016/j.jpeds.2019.02.009 30905424

[B19] Hamamoto Y. Ano S. Allard B. O’Sullivan M. McGovern T. K. Martin J. G. (2017). Montelukast reduces inhaled chlorine triggered airway hyperresponsiveness and airway inflammation in the mouse. Br. J. Pharmacol. 174, 3346–3358. 10.1111/bph.13953 28718891 PMC5595758

[B20] Han J. Jia Y. Takeda K. Shiraishi Y. Okamoto M. Dakhama A. (2010). Montelukast during primary infection prevents airway hyperresponsiveness and inflammation after reinfection with respiratory syncytial virus. Am. J. Respir. Crit. Care Med. 182, 455–463. 10.1164/rccm.200912-1811OC 20442434 PMC2937239

[B21] Hareedy M. S. Ahmed E. A. Ali M. F. (2019). Montelukast modifies simvastatin-induced myopathy and hepatotoxicity. Drug Dev. Res. 80, 1000–1009. 10.1002/ddr.21581 31389048

[B22] Hormati A. Ahmadpour S. Afkhami Ardekani M. Khodadust F. Refahi S. (2020). Radioprotective effects of montelukast, a selective leukotriene CysLT1 receptor antagonist, against nephrotoxicity induced by gamma radiation in mice. J. Biochem. Mol. Toxicol. 34, e22479. 10.1002/jbt.22479 32125029

[B23] Hoxha M. Lewis-Mikhael A. M. Bueno-Cavanillas A. (2018). Potential role of leukotriene receptor antagonists in reducing cardiovascular and cerbrovascular risk: A systematic review of human. Biomed. Pharmacother. 106, 956–965. 10.1016/j.biopha.2018.07.033 30119268

[B24] Huynh T. Wang H. Luan B. (2020). *In Silico* Exploration of the Molecular Mechanism of Clinically Oriented Drugs for Possibly Inhibiting SARS-CoV-2’s Main Protease. J. Phys. Chem. Lett. 11, 4413–4420. 10.1021/acs.jpclett.0c00994 32406687

[B25] Khomich O. A. Kochetkov S. N. Bartosch B. Ivanov A. V. (2018). Redox Biology of Respiratory Viral Infections. Viruses 10, 392. 10.3390/v10080392 PMC6115776 30049972

[B26] Kimura H. Yoshizumi M. Ishii H. Oishi K. Ryo A. (2013). Cytokine production and signaling pathways in respiratory virus infection. Front. Microbiol. 4, 276. 10.3389/fmicb.2013.00276 24062733 PMC3774987

[B27] Kloepfer K. M. DeMore J. P. Vrtis R. F. Swenson C. A. Gaworski K. L. Bork J. A. (2011). Effects of montelukast on patients with asthma after experimental inoculation with human rhinovirus 16. Ann. Allergy Asthma. Immunol. 106, 252–257. 10.1016/j.anai.2010.11.021 21354028 PMC3104731

[B28] Klok F. A. Kruip M. J. H. A. van der Meer N. J. M. Arbous M. S. Gommers D.A.M.P.J. Kant K. M. (2020). Incidence of thrombotic complications in critically ill ICU patients with COVID-19. Thromb. Res. 191, 145–147. 10.1016/j.thromres.2020.04.013 32291094 PMC7146714

[B29] Korkmaz K. Gedik H. S. Budak A. B. Yener A. U. Kaya E. Genc S. B. (2015). Effect of Montelukast on Spinal Cord Ischemia- Reperfusion Injury. Turk. Neurosurg. 25, 757–765. 10.5137/1019-5149.JTN.11499-14.2 26442542

[B30] Kotchetkov R. McLean J. Nay D. Gerard L. Hopkins S. Didiodato G. (2020). Premedication with montelukast and rupatadine decreased rituximab infusion time, rate, severity of reactions and use of rescue medications. Int. J. Cancer 147, 1979–1986. 10.1002/ijc.32985 32189328

[B31] Landeras-Bueno S. Fernández Y. Falcón A. Oliveros J. C. Ortín J. (2016). Chemical Genomics Identifies the PERK-Mediated Unfolded Protein Stress Response as a Cellular Target for Influenza Virus Inhibition. mBio 7, e00085–e00e16. 10.1128/mBio.00085-16 27094326 PMC4850254

[B32] Lenz Q. F. Arroyo D. S. Temp F. R. Poersch A. B. Masson C. J. Jesse A. C. (2014). Cysteinyl Leukotriene Receptor (CysLT) Antagonists Decrease Pentylenetetrazol-Induced Seizures and Blood-Brain Barrier Dysfunction. Neuroscience 277, 859–871. 10.1016/j.neuroscience.2014.07.058 25090924

[B33] Mao L. Jin H. Wang M. Hu Y. Chen S. He Q. (2020). Neurologic Manifestations of Hospitalized Patients With Coronavirus Disease 2019 in Wuhan, China. JAMA Neurol. 77, 1–9. 10.1001/jamaneurol.2020.1127 PMC7149362 32275288

[B34] Marschallinger J. Schäffner I. Klein B. Gelfert R. Rivera F. J. Illes S. (2015). Structural and functional rejuvenation of the aged brain by an approved anti-asthmatic drug. Nat. Commun. 6, 8466. 10.1038/ncomms9466 26506265 PMC4639806

[B35] Miwa N. Nagano T. Ohnishi H. Nishiuma T. Takenaka K. Shirotani T. (2018). An Open-Label, Multi-Institutional, Randomized Study to Evaluate the Additive Effect of a Leukotriene Receptor Antagonist on Cough Score in Patients with Cough-Variant Asthma Being Treated with Inhaled Corticosteroids. Kobe. J. Med. Sci. 64, E134–E139. 30728339 PMC6347040

[B36] Morita Y. Campos Alberto E. Suzuki S. Sato Y. Hoshioka A. Abe H. (2017). Pranlukast reduces asthma exacerbations during autumn especially in 1- to 5-year-old boys. Asia. Pac. Allergy 7, 10–18. 10.5415/apallergy.2017.7.1.10 28154801 PMC5287065

[B37] Mueller C. F. Becher M. U. Zimmer S. Wassmann S. Keuler B. Nickenig G. (2010). Angiotensin II triggers release of leukotriene C4 in vascular smooth muscle cells via the multidrug resistance-related protein 1. Mol. Cell. Biochem. 333, 261–267. 10.1007/s11010-009-0227-x 19685171

[B38] Oral A. Odabasoglu F. Halici Z. Keles O. N. Unal B. Coskun A. K. (2011). Protective effects of montelukast on ischemia-reperfusion injury in rat ovaries subjected to torsion and detorsion: biochemical and histopathologic evaluation. Fertil. Steril. 95, 1360–1366. 10.1016/j.fertnstert.2010.08.017 20850724

[B39] Peng J. Zhou H. Kuang G. Xie L. Tian T. Liu R. (2017). The selective cysteinyl leukotriene receptor 1 (CysLT1R) antagonist montelukast regulates extracellular matrix remodeling. Biochem. Biophys. Res. Commun. 484, 474–479. 10.1016/j.bbrc.2017.01.052 28088523

[B40] Ruiz I. Nevers Q. Hernández E. Ahnou N. Brillet R. Softic L. (2020). MK-571, a cysteinyl leukotriene receptor-1 antagonist, inhibits hepatitis C virus (HCV) replication. Antimicrob. Agents. Chemother. 64, e02078–e02019. 10.1128/AAC.02078-19 32179525 PMC7269486

[B41] Russell B. Moss C. George G. Santaolalla A. Cope A. Papa S. (2020). Associations between immune-suppressive and stimulating drugs and novel COVID-19-a systematic review of current evidence. Ecancermedicalscience 14, 1022. 10.3332/ecancer.2020.1022 32256705 PMC7105343

[B42] Sayin T. Cimen S. Cimen S. Bostanci T. Akbaba S. Yildirim Z. (2020). Colonic anastomosis can be protected from ischemia reperfusion injury with intra-peritoneal Montelukast treatment. Asian. J. Surg. 43, 130–138. 10.1016/j.asjsur.2019.01.022 30948265

[B43] Shao H. Lan D. Duan Z. Liu Z. Min J. Zhang L. (2006). Upregulation of mitochondrial gene expression in PBMC from convalescent SARS patients. J. Clin. Immunol. 26, 546–554. 10.1007/s10875-006-9046-y 17024565 PMC7086694

[B44] Sılay M. S. Toklu H. Özağarı A. Aydın M. Tetik Ş. Şener G. (2014). Montelukast prevents testes against ischemia-reperfusion injury through suppression of iNOS expression. Turk. J. Urol. 40, 221–227. 10.5152/tud.2014.61587 26328182 PMC4548366

[B45] Topaloğlu N. Olgun Yıldızeli Ş. Şener G. Laçin T. Şehirli Ö. Bozkurtlar E. (2018). Protective effect of cysteinyl leukotriene receptor antagonist montelukast in bleomycin-induced pulmonary fibrosis. Turk. Gogus. Kalp. Damar. Cerrahisi. Derg. 26, 588–597. 10.5606/tgkdc.dergisi.2019.15149 32082801 PMC7018181

[B46] van den Brand J. M. Haagmans B. L. van Riel D. Osterhaus A. D. Kuiken T. (2014). The pathology and pathogenesis of experimental severe acute respiratory syndrome and influenza in animal models. J. Comp. Pathol. 151, 83–112. 10.1016/j.jcpa.2014.01.004 24581932 PMC7094469

[B47] Varga Z. Flammer A. J. Steiger P. Haberecker M. Andermatt R. Zinkernagel A. S. (2020). Endothelial cell infection and endotheliitis in COVID-19. Lancet 395, 1417–1418. 10.1016/S0140-6736(20)30937-5 32325026 PMC7172722

[B48] Wang K. Birring S. S. Taylor K. Fry N. K. Hay A. D. Moore M. (2014). Montelukast for postinfectious cough in adults: a double-blind randomised placebo-controlled trial. Lancet Respir. Med. 2, 35–43. 10.1016/S2213-2600(13)70245-5 24461900

[B49] Wang H. Cheng Y. Liu Y. Shi J. Cheng Z. (2019). Montelukast promotes mitochondrial biogenesis via CREB/PGC-1α in human bronchial epithelial cells. Artif. Cells Nanomed. Biotechnol. 47, 4234–4239. 10.1080/21691401.2019.1687502 31722576

[B50] Wang Y. Tian J. Liu J. (2020). Suppressive Effect of Leukotriene Antagonists on Capsular Contracture in Patients Who Underwent Breast Surgery with Prosthesis: A Meta-Analysis. Plast. Reconstr. Surg. 145, 901–911. 10.1097/PRS.0000000000006629 32221199

[B51] Wu A. Y. Chik S. C. Chan A. W. Li Z. Tsang K. W. Li W. (2003). Anti-inflammatory effects of high-dose montelukast in an animal model of acute asthma. Clin. Exp. Allergy 33, 359–366. 10.1046/j.1365-2222.2003.01615.x 12614451

[B52] Yeh D. Y. Yang Y. C. Wang J. J. (2015). Hepatic Warm Ischemia-Reperfusion-Induced Increase in Pulmonary Capillary Filtration Is Ameliorated by Administration of a Multidrug Resistance-Associated Protein 1 Inhibitor and Leukotriene D4 Antagonist (MK-571) Through Reducing Neutrophil Infiltration and Pulmonary Inflammation and Oxidative Stress in Rats. Transplant. Proc. 47, 1087–1091. 10.1016/j.transproceed.2014.10.061 26036526

[B53] Zamani M. Prabhakaran M. P. Varshosaz J. Mhaisalkar P. S. Ramakrishna S. (2016). Electrosprayed Montelukast/poly (lactic-co-glycolic acid) particle based coating: A new therapeutic approach towards the prevention of in-stent restenosis. Acta Biomater. 42, 316–328. 10.1016/j.actbio.2016.07.007 27397493

[B54] Zhang X. Tan Y. Ling Y. Lu G. Liu F. Yi Z. (2020). Viral and host factors related to the clinical outcome of COVID-19. Nature 583, 437–440. 10.1038/s41586-020-2355-0 32434211

[B55] Zhou X. Cai J. Liu W. Wu X. Gao C. (2019). Cysteinyl leukotriene receptor type 1 (CysLT1R) antagonist zafirlukast protects against TNF-α-induced endothelial inflammation. Biomed. Pharmacother. 111, 452–459. 10.1016/j.biopha.2018.12.064 30594784

[B56] Zhou L. Sun X. Shi Y. Liu J. Luan G. Yang Y. (2019). Cysteinyl leukotriene receptor type 1 antagonist montelukast protects against injury of blood-brain barrier. Inflammopharmacology 27, 933–940. 10.1007/s10787-019-00611-7 31313075

